# Editorial: Women in elite sports and performance enhancement: 2021

**DOI:** 10.3389/fspor.2022.999969

**Published:** 2022-09-09

**Authors:** Louise Deldicque

**Affiliations:** Institute of Neuroscience, UCLouvain, Louvain-la-Neuve, Belgium

**Keywords:** female athlete, female scientist, coaching, lipid profile, concussion sport, telomere length, 9 cross-country ski, netball 10

## Introduction

According to the UNESCO Institute for Statistics, less than 30% of the world's researchers are women. In sports science and sports medicine research, underrepresentation and under-participation of women have been a matter of debate for a long time now (Naik et al., 2022). However, it is only recently that the field has recognized the imbalance between genders and taken measures to overcome this unfair situation. One representative example of this is the present Research Topic aiming at encouraging women scientists to submit a manuscript as the first or last author. In addition to the underrepresentation of women in sports research, the percentage of female participants in sports science studies averages 30 to 40% of overall participants, with only 6% of studies investigating exclusively women (Cowley et al., 2021). This is very striking and questionable, especially as the number of women participating in sporting events is growing. Finally, another issue is the low number of female athletes, trainers, coaches, and decision-makers, which is even more glaring in elite sport (Mujika and Taipale, 2019). It is now time to take action to nurture the participation of women in sports science and sports medicine research.

## This issue

The present issue contributes to the reduction of the gap between men and women in sports sciences by stimulating women scientists to publish their work as well as reporting data obtained from female athletes. Authors were primarily women, namely 18 on 24, or 75% of authors were women. In addition, out of the 496 subjects included in the 5 studies published in this special issue, 259 were female, which represents 52% of the subjects.

The purpose of the first study was to develop a live and flexible normative dataset for netball (Croft et al.). These normative data tables would provide coaches with standards for assessing the performance of players, across competition levels against the player positions. The second study aimed at comparing differences in race time between female and male paraplegic and able-bodied skiers and the winner and at investigating whether those differences change across seasons (Carlsen et al.). Paraplegic and female skiers displayed larger variability in performance than able-bodied and male skiers, respectively, and those differences were relatively stable across the 2011–2020 seasons. The third study investigated the impact of concussion, sport, and time in season on saliva telomere length in 108 male and 75 female athletes from different disciplines (Machan et al.). No associations were found between telomere length and history of concussion, age, or sport contact type while telomere length was larger mid- compared to pre-season as well as in men compared to women. Thus, from a practical point of view, both time in season and sex should be considered when evaluating telomere length in saliva as a possible biomarker for sport-related concussions. The fourth study examined the lipid profiles of 78 female student-athletes participating at different organized sports competition levels (Vento et al.). Confirming the initial hypothesis, total cholesterol, and total cholesterol to high-density lipoprotein ratio were both within a healthy recommendation rate, independently of the competition level. It was concluded that regardless of competition level, organized sports participation may help meet physical activity requirements to promote a physically active lifestyle, support healthy lipid profiles, and prevent cardiovascular disease in female college students. The fifth and last study synthesized and summarized the current challenges and barriers women sports coaches face in Australia (Roberts et al.). A lack of understanding of the current figures and roles of women coaches is pointed out, which should be first fixed before looking for a better understanding of the context-specific barriers and facilitators that women coaches face in their careers.

## Perspectives

The present Research Topic contributes modestly to the participation of women scientists in sports science and sports medicine research. This is an important step toward a reduction in the imbalance between male and female authors in the field. Considering that more and more women engage in physical activity programs and participate in sports events, it is of the utmost importance to give them sex- and gender-specific advice and support. And whom better than women can understand the issues women may encounter while practicing sport? In the near future, it will be critical for the sports sciences and sport medicine research to recognize and constrain implicit gender bias to stimulate women's representation and participation (Bekker et al., 2018). Much has been done during the last few years, but there is still much space for improvement.

## Author contributions

The author confirms being the sole contributor of this work and has approved it for publication.

## Conflict of interest

The author declares that the research was conducted in the absence of any commercial or financial relationships that could be construed as a potential conflict of interest.

## Publisher's note

All claims expressed in this article are solely those of the authors and do not necessarily represent those of their affiliated organizations, or those of the publisher, the editors and the reviewers. Any product that may be evaluated in this article, or claim that may be made by its manufacturer, is not guaranteed or endorsed by the publisher.
